# Peer assisted learning in the clinical setting: an activity systems analysis

**DOI:** 10.1007/s10459-014-9557-x

**Published:** 2014-10-01

**Authors:** Deirdre Bennett, Siun O’Flynn, Martina Kelly

**Affiliations:** 1 Medical Education Unit, School of Medicine, Brookfield Health Sciences Complex, University College Cork, Cork, Ireland; 2 Department of Family Medicine, University of Calgary, Calgary, Canada

**Keywords:** Activity systems analysis, Cultural historical activity theory, Sociocultural theory, Peer assisted learning, Clinical learning environment, Graduate entry medicine, Medical education

## Abstract

Peer assisted learning (PAL) is a common feature of medical education. Understanding of PAL has been based on processes and outcomes in controlled settings, such as clinical skills labs. PAL in the clinical setting, a complex learning environment, requires fresh evaluation. Socio-cultural theory is proposed as a means to understand educational interventions in ways that are practical and meaningful. We describe the evaluation of a PAL intervention, introduced to support students’ transition into full time clinical attachments, using activity theory and activity systems analysis (ASA). Our research question was *How does PAL transfer to the clinical environment?* Junior students on their first clinical attachments undertook a weekly same-level, reciprocal PAL activity. Qualitative data was collected after each session, and focus groups (n = 3) were held on completion. Data was analysed using ASA. ASA revealed two competing activity systems on clinical attachment; Learning from Experts, which students saw as the primary function of the attachment and Learning with Peers, the PAL intervention. The latter took time from the first and was in tension with it. Tensions arose from student beliefs about how learning takes place in clinical settings, and the importance of social relationships, leading to variable engagement with PAL. Differing perspectives within the group were opportunities for expansive learning. PAL in the clinical environment presents challenges specific to that context. Using ASA helped to describe student activity on clinical attachment and to highlight tensions and contradictions relating PAL in that setting. Planning learning opportunities on clinical placements, must take account of how students learn in workplaces, and the complexity of the multiple competing activity systems related to learning and social activities.

## Introduction

Peer assisted learning (PAL) can be defined as *People from similar social groupings, who are not professional teachers, helping each other to learn and learning themselves by teaching* (Topping 1996). The key element is that peer teachers are neither content nor teaching experts (Ross and Cameron 2007). PAL has become a common feature of medical education (Ross and Cameron 2007; Salerno-Kennedy et al. 2010; Ten Cate and Durning 2007b) and its benefits are well described (Ten Cate and Durning 2007b), for both peer teacher and learner. The cognitive and social congruence between peers has been shown to promote delivery of appropriately pitched teaching in a safe environment (Bulte et al. 2007). Peer teachers, because of their proximity to the learner, are more likely to understand which aspects of a topic learners may find conceptually difficult and to explain it in ways that are easily understood. The social proximity of peer teacher and learner is thought to allow students to express difficulties more comfortably, to feel relaxed and build confidence through observation of a peer in a teaching role. Development of organisational skills, communication/presentation skills (Hill et al. 2010), confidence, leadership (Secomb 2008) and improved assessment performance (Peets et al. 2009) are amongst the benefits to peer teachers.

In medical education, PAL programmes are often characterised by formal structures and processes. Typically, PAL interventions take place in controlled settings (Batchelder et al. 2010; Ten Cate 2007; Glynn et al. 2006; Ross and Cameron 2007), such as clinical skills labs. The theme and content of such teaching is usually pre-determined (Batchelder et al. 2010) Those given the peer -teacher role may be selected on the basis of prior academic performance and may undergo specific training in the material to be delivered and/or in teaching skills (Ten Cate 2007; Bulte et al. 2007). Understanding of PAL interventions to date is based on the process and outcomes seen in these controlled circumstances (Pasquale and Cukor 2007; Peets et al. 2009). Introduction of PAL to the clinical setting seems a natural progression from clinical skills labs. Much of the learning that happens in clinical settings at undergraduate and postgraduate level is facilitated informally by those a few years ahead of the learner. However, transfer of more formal PAL to the clinical environment presents challenges. The clinical workplace affords and constrains learning, in ways that are variable and difficult to predict. Learning is one, of many, competing activities simultaneously taking place in clinical settings. It is more opportunistic and often less structured than that taking place in classroom or skills lab settings, relying on the availability of suitable patients and time for faculty to provide appropriate support. There have been few studies of PAL in clinical settings (Nikendei et al. 2009; Schauseil-Zipf et al. 2010; Heckmann et al. 2008). All were controlled, outcomes based studies involving near peer teaching of specific clinical skills on the ward as part of a clinical placement. While Nikendei et al. (2009) and Schauseil-Zipf et al. (2010) found that self- assessed clinical competence was enhanced by PAL, in comparison with students taught by faculty alone, Heckmann et al. (2008) found that PAL was as effective as faculty teaching, rather than better, when measured by scores in written tests and OSCE, as well as self- assessed competence. Schauseil-Zipf et al. (2010) reported high levels of student satisfaction with the process. However, more clarification research is needed. Introducing PAL into the clinical environment represents a significant change of context that requires re-evaluation of the process.

We describe the evaluation of a same-level reciprocal PAL intervention, in the clinical setting. The intervention was introduced to support students in their transition into full time clinical attachments. Undergraduate clinical attachments in the UK and Ireland differ from those in North America in terms of the degree to which students are assimilated into the clinical team and are responsible for patient care. Our students typically attend ward rounds, outpatient clinics and theatre with their team, as observers. They take histories and examine patients independently or in pairs. There are scheduled sessions of teaching delivered by faculty, both at the bedside and in tutorial rooms. Self- direction is an important aspect of clinical attachments, as there may be significant time spent apart from the team. Students have been shown to benefit from organisational, pedagogic and pastoral support during this transition period (Dornan et al. 2005) as self-direction in a complex and unfamiliar environment is difficult. Feedback from our students indicated that they felt that more structure was needed during times when they were expected to direct their own learning. The introduction of PAL sessions to clinical attachments offered an opportunity to address this need, to provide organisational support by scheduling a structured activity, and to provide pedagogic support by encouraging students to take responsibility for their own learning. Our over-arching objective was to support students’ development as a community of learners, by providing a framework within which students could work together, to encourage mutual engagement amongst students and to promote the development of domain independent skills (Ten Cate and Durning 2007b; Dandavino et al. 2007) such as teamwork, organisation and communication skills.

### Conceptual orientation

In this study we take a socio-cultural approach to evaluation of PAL. Socio-cultural theories of learning have been proposed as an alternative to outcomes based approaches, as a means to understand educational interventions (Mann 2011; Swanick 2005), in ways that are practical and meaningful for stakeholders (Hodges and Kuper 2012). Socio-cultural theory emphasises learning through participation in social activity, in cultural and historical contexts (Bleakley 2010). From a socio-cultural perspective, learning and the context in which it takes place are inseparable. Learning in medical workplaces is ideally suited to this orientation, as medical students learn while they participate, peripherally, in the practice of medicine (Lave and Wenger 1991). The ways in which patient care is delivered, and therefore how students learn, are embedded in historical and cultural contexts. Context shapes the way learning happens and vice versa. Socio-cultural theory treats the context and the activity (learning at work) as a single unit of analysis (Yamagata-Lynch 2010). What is understood about PAL in controlled settings cannot, therefore, be assumed to apply in a more complex learning environment. Focussing on educational processes, using a socio-cultural theoretical lens, allows researchers to capture learning in context, identifying unanticipated consequences. This is an advantage over outcomes based studies which examine anticipated outcomes only.

Communities of practice (COP) (Lave and Wenger 1991) theory is perhaps the most frequently cited socio-cultural theory in the medical education literature. It describes how learning happens and identities are formed in communities brought together by shared practices in a particular domain (ibid). A less frequently cited, socio-cultural theory, is activity theory (Engestrom 1993), arising from the work of Vygotsky (1978), Leontiev (1981). Activity theory also focusses on social practices or activities but describes, in particular, how multiple activities taking place within a complex environment, interact with each other to create opportunities for learning. COP theory emphasises learning through shared practice, although it also describes the value of learning at boundaries between communities. In activity theory the learning is in the conflict between concurrent activities, which is similar to the notion of learning at boundaries. We chose to use activity theory and its associated methodology, activity systems analysis (ASA) (Yamagata-Lynch 2010), in our evaluation of PAL in the clinical setting, because we were introducing a new activity to an existing learning environment and wanted to assess its impact on our students’ activity within that environment.

**Fig. 1:**
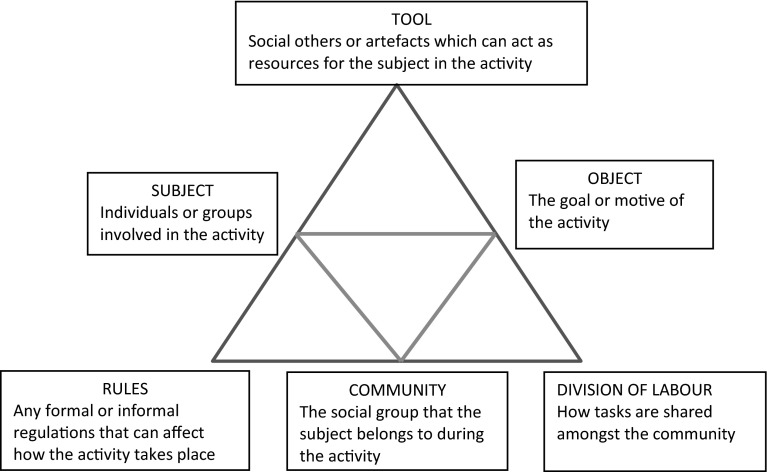
Triangular diagram showing the elements of an activity system

The basic tenet of activity theory is that human activity is object oriented, mediated by tools and socio-culturally situated (Vygotsky 1978) In other words, goals are achieved by social activity. We work towards these goals through the use of tools, material and abstract, and our activity is shaped by the social, cultural and historical environment in which it occurs. Leontiev (1981) provides an example of goal oriented social activity in his description of the primeval hunt, where there is a shared understanding between hunters that the goal is to kill the prey so that it can be converted to food and clothing to meet the needs of the individual and the collective. Some of the group frighten the prey, others catch and kill it but they are united and guided by the goal of the activity, which gives meaning to their actions and the actions of others (Leontiev 1981). ASA attempts to capture a view of activity in context and to communicate complex findings through triangular diagrams (Engestrom 2009) (Fig. 1). Within the scenario of the primeval hunt, the hunters are the subjects of the activity, and the prey is the object. The object in activity theory is that which is transformed by the activity but is also the motivation for the activity. There are a variety of tools used in this activity, physical tools for hunting and killing, but also “thinking tools” or shared understandings, of how hunting is conducted. The “labour” of hunting is divided amongst the group and “rules” of hunting are followed, in ways that are culturally and historically derived.

Engestrom further developed activity theory to capture multiple activities occurring in the same setting simultaneously, and the relationships between these (Engestrom 2009). In this instance, triangular diagrams are used to identify tensions and contradictions within and between multiple competing activity systems in one environment. Change within a system, such as the introduction of a new tool or activity, may bring new tensions, within or between activity systems. Contradictions and tensions between activities are not necessarily negative and can be drivers for growth and change within the system (Doll and Trueit 2010). Such contradictions may provide the basis for “expansive” learning, where culturally new patterns of activity are produced (Engestrom 2009) as a result of conflicting demands placed on subjects in context.

We chose to use activity theory because it is ideally suited to exploring complex living systems, such as clinical learning environments, because of its compatibility with complexity thinking (Bleakley 2010). Complexity thinking recognises that living systems are ever changing, evolving and transforming. Activities within complex systems interact in dynamic, non-linear ways. Activity theory, is particularly suited to describing real-world learning and finding solutions to complex work problems (Yamagata-Lynch 2010). It has been used to examine learning about patient safety in clinical environments during the transition from undergraduate to postgraduate training (de Feijter et al. 2011). ASA allows researchers to find systemic implications and to understand systemic contradictions and tensions.

Our research question was

How does Peer Assisted Learning transfer to the clinical environment?

## Methodology

### Participants

This study took place in the School of Medicine, University College Cork, Ireland in 2010–2011 amongst students undertaking their first full time hospital placements. The students are a diverse group, of Graduate (GE) and “Direct” (DE) entrants. Graduate entrants hold a prior degree, while the majority of Direct entrants are school leavers, with a small number of Mature (>23 years) students. During the term in which this study was undertaken, Graduate Entry Year 2 and Direct Entry Year 3 classes come together for full time clinical placements, having pursued separate programmes up to that point. Some joint teaching and activities occur in the years prior to merging. Subsequently, they remain as a single cohort for the remainder of the programme. The Direct Entry group constitute 70 % of the cohort. Students rotate through two hospitals, typically a tertiary referral centre and a smaller general hospital site, spending 4 weeks at each site.

Both programmes feature integrated systems based curricula with early clinical exposure and student led, faculty facilitated case based learning. Students therefore have experience of leading small group sessions, using clinical cases to generate learning objectives, and presenting and discussing their work with each other, albeit with a faculty member present. The Peer Mini Clinical Examination is already an established part of the curriculum during this year of the program (Bennett et al. 2012), with students undertaking a reciprocal peer assessment at the end of each hospital attachment. Prior to commencement of this PAL programme, students were introduced to the objectives and benefits of PAL and given specific instruction regarding the process they were to undertake. Students were trained in giving and receiving feedback and on presentation skills. Documents outlining learning objectives and guidance on how to run the sessions were provided through our Virtual Learning Environment and PAL sessions were supported at the site by clinical site staff.

#### Intervention

The intervention was a weekly reciprocal PAL session in the clinical setting. We designed the PAL sessions as student led case based discussions because this was a format familiar to our students, it anchored the sessions to their clinical experiences and because case based discussion has been shown to be a successful format for PAL (Nelson et al. 2013) By incorporating the identification of learning objectives linked to cases seen on placement we aimed to support greater self- direction amongst our students. Students were allocated to groups of 4 or 5 peers for PAL sessions, which were to occur once per week. Students rotated the role of peer teacher weekly. The peer teacher presented the case of a patient seen on the wards. The group provided feedback to the peer teacher on their presentation. The peer teacher then taught on a topic related to the case. Students selected the topic for the session themselves, as an important learning objective linked to the case. They were also guided in their choice of topic by curricular documents. Students had already received teaching in core clinical topics and therefore, review and application of prior learning to real cases was the focus of the PAL sessions. These sessions took place at the bedside and in other locations within the clinical sites. Each student attended a total of 7 PAL sessions. Participation in the program did not contribute to student evaluation as this was a pilot project.

## Data collection and analysis

One hundred and fifteen students took part in the PAL sessions across 4 hospital sites. Demographics are shown in Table 1.

**Table 1:** Student demographics

	Graduate entry	Direct entry
N	35	80
Mean age (range)	25.7 (23–36)	21.4 (19–44)
Gender	57 % male (n = 20)	54 % female (n = 43)

After each session, every student was asked to provide information about the session, topic, location, duration etc. and written qualitative feedback in response to two open questions; what was good about the session and what could be improved. Return of the feedback forms was incentivised by entry into a raffle for a small cash prize (50 euro). The student returning the form was not anonymous, to allow inclusion in the raffle, however the peer teacher for the session evaluated was not named on the form.

Three focus groups (n = 8–10 in each) were held at the end of the year. Students were purposively selected to ensure that both Direct and Graduate Entry students and all clinical sites attended were represented. The focus groups were 1 h in length and were facilitated by DB & MK. DB & MK are both medical school faculty members with roles as year co-ordinators. Both are doctors who hold an educational qualification and are experienced facilitators of focus groups. Students were asked to discuss their experience of the PAL intervention and how they felt it contributed to their learning. Students consented to the recording of the discussions.

Observations of PAL sessions were not conducted as it was felt that faculty presence would significantly affect the manner in which the sessions were conducted.

Transcriptions of the focus group discussions were imported into NVivo 9 (QSR International), as were the written comments. The researchers immersed themselves in the data and identified and collectively agreed the mediated activities described by participants. A thematic content analysis was then performed on all transcribed data by DB & MK. Each text was examined systematically by identifying and grouping themes and coding. Following preliminary independent analysis DB & MK met to discuss initial coding and refine definitions. As data analysis progressed, the researchers met to compare emerging issues, resolve discrepancies and categorise codes into larger themes. SO’F independently reviewed the data and contested themes. Thick descriptions of the activities identified were developed. The themes relating to each of the previously identified activity systems were then mapped to the headings shown in Table 1 above, for example object, tools etc. (Yamagata-Lynch 2010) to allow communication of findings using the triangular diagrams (Mwanza 2002). Tensions/contradictions between elements of the concurrent activities were identified independently by DB & MK, and then agreed through reflexive discussion amongst all authors. Results were reported to students via email and responses invited.

Ethical approval for this study was granted by the Clinical Research Ethics Committee of the Cork Regional Hospitals.

## Results

146 teaching sessions (80 % of full participation) took place over a period of 16 weeks in total. 33 occurred at the bedside & 113 elsewhere in the clinical site, tutorial rooms, wards and cafeteria. The sessions covered history taking, examination and content knowledge on a wide variety of topics. The duration was 30 min on average. A total of 590 written feedback forms were returned. Responses varied from a few words to half an A4 page in length. Students made many positive comments regarding the teaching sessions conducted by their peers.

**Fig. 2:**
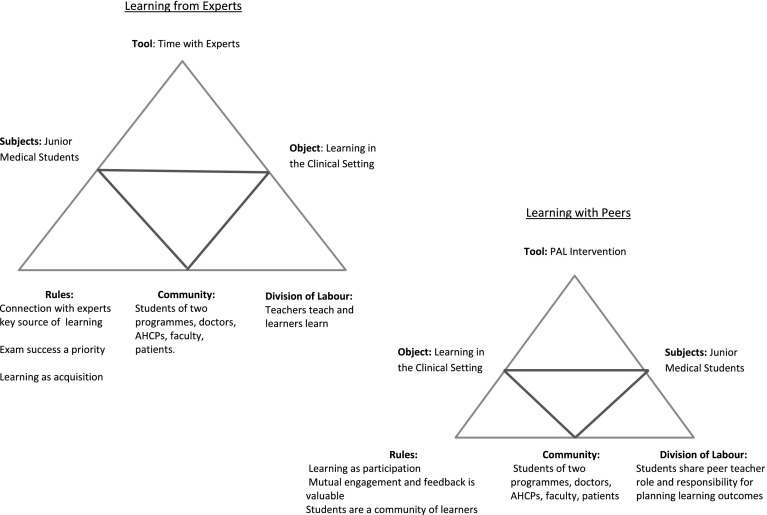
Activity systems in the clinical setting

Two competing activity systems were identified involving students on clinical attachment. Triangular diagrams are seen in Fig. 2 illustrating these. The first is entitled Learning from Experts. This was the activity which students saw as the primary function of their clinical attachments. The Learning with Peers, through the PAL intervention, represented a second competing activity, which took time from the first and was in tension with it. The *subject* in both systems is the medical student and the *object* is learning in the clinical setting. The *tool,* for Learning from Experts, is time spent with experts, while the tool for Learning with Peers is the PAL process. The *community* in which both activities took place was the hospital community, comprising patients, fellow students, university faculty, junior doctors, consultants, nurses and other allied health care professionals. The *rules* and *division of labour* for the PAL process were prescribed by faculty. We now discuss the rules under which both activities took place, the division of labour within them and the tensions/contradictions between them (see Fig. 2).

### Rules

#### Connection with experts

Theories of workplace learning (Lave and Wenger 1991; Billett 2006a) emphasise the importance of access to senior figures for novices in the workplace. Listening to the ways that experts talk, hearing their stories and their experiences and being recognised by them as a legitimate community member are central to professional identity formation (Lave and Wenger 1991). This desire to connect with experts was strong amongst our students and drove Learning from Experts as the dominant activity on clinical attachment.

Comparing the experience of being taught by a senior clinician with being taught by peers, one student commented;

The consultant told us the 5 questions you must ask every patient who is short of breath - that is experience.. that is not going to be in a book anywhere. A student is going to say these are the 55 questions we must ask. Focus Group 1. DE Student, Male.

A similar statement was made by another student

People who have gone through it before can kind of put it into a better perspective for some reason and summarise….. these are the signs, these are the 3 things you look for Focus Group 2. DE Student, Male.

In both of these quotes, we see that students recognise the value of expert experience in the application of knowledge to practice. This expertise is hard to access, it may not be in books. It is not something students feel they might work out together themselves. This focus on capturing the expertise of senior figures is in tension with the PAL tool, in use in the Learning with Peers activity, which is underpinned by the ideas of self- direction and connection with non-experts.

#### Exam focus

There was a strong theme of exam focussed learning amongst students.

The case itself, although interesting, is not a fundamental part of the fast approaching OSCE Feedback Form, DE Student, Female.

Perceived relevance of knowledge to the curriculum, within the academic year or even in a particular hospital placement, also defined its value. One student, referring to abdominal aortic aneurysm, stated (incorrectly);

The topic is not very relevant to what we need to know in third year. Feedback Form DE Student, Male.

The desired outcome of the Learning with Peers activity, which was not content focussed but emphasised mutual engagement and teamwork, was in tension with students’ “rule” that learning is about the acquisition of knowledge for exams.

#### Social relationships & collaborative learning

This intervention was designed on the understanding that students would be willing to provide each other with constructive feedback. Many students, however, were reluctant to compromise their social relationships for the sake of collaborative learning. This was particularly evident in relation to giving feedback.

I just find it difficult to give negative feedback just to your classmates, you are afraid they will take it badly… it is kind of difficult to say negative things Focus Group 2, DE Student, Female.

Views varied as to whether it was easier to give feedback to friends or to those you don’t know as well. However, it was clear that the merging of the Direct Entry and Graduate Entry cohorts, in particular, meant that students were keen to manage the impressions of the other group.

The last thing you want to do is foster difference….. and you don’t want to burn bridges early by giving some really bad feedback Focus Group 1, GE Student, Female.

#### Tension within the student group

There was tension evident amongst some of the students in relation to attitudes to the PAL process. Some Graduate Entry students found that the school leaver group were less likely to engage with the activity and this was a source of frustration.

I was with 3 Direct Entry students who were younger than me,…..I could see the benefit of it, and I could see that it would be in our future career,… and I enjoyed it but the other 3 people I was with really just saw it as a thing to get out of the way. Focus Group 1, GE student, Male.

The lack of effort invested by some school leaver students was a recurrent theme.

I found a lot of them (school leavers) just weren’t nearly as enthusiastic about doing it as the rest of the graduates that I was around with… so it kind of made it a little bit frustrating to put a lot of work or effort into it, when they seemed like they just didn’t want to do it. Focus Group 1, GE student, Male

In one focus group, when school leaver students put forward the view that they were not ready for the responsibility of engaging with PAL without tutor supervision, this was challenged by a graduate student.

There is something to be said for giving us the opportunity to actually say, let’s run this ourselves, let’s think about this ourselves, let’s criticise ourselves and I think a lot of people are failing to do that right now. But I think we need to make sure that we don’t continue to fail at that, because this is an opportunity for us Focus Group 2, GE Student, Male.

This tension within the student group may for the basis for expansive learning.

#### Tension between “Learning from Experts” and “Learning with Peers” Activities

Time is limited on clinical attachments and access to experts is also limited. There was a tension therefore between Learning from Experts and spending time Learning with Peers.

It was difficult to organise it as we have exams coming up and have lots of study to do and were very busy. Feedback Form, DE Student, Female.

The PAL process was often viewed as a distraction from “proper” learning.

It was a distraction from rounds with our teams and we missed vital learning opportunities. Feedback Form DE Student, Male.

Learning with peers, for many students, is low priority, something to be squeezed in around other “better” learning.

Everybody thought in the back of their mind that this wasn’t great value when you weren’t marked for it Focus Group 3, DE Student, Female.

Even for students who could see potential benefits to the concept of PAL, this assessment driven attitude prevailed.

It is a really good idea in theory but when it is not worth any marks or anything like the enthusiasm for it isn’t going to be there Focus Group 2, GE Student, Male.

## Division of labour

In the Learning from Experts activity system, students feel comfortable with their role as novice/learner and with the role of the expert as source of knowledge and experience. Roles and responsibilities were assigned to the students for the PAL exercise. Roles of peer teacher, student and evaluator of the PAL process, were rejected by many, as illustrated by variable engagement with PAL & desire for faculty supervision.

### Discomfort in the peer teacher role

The discomfort some students felt in the peer teacher role is reflected in the following quotes.

Not that comfortable with the leadership role Feedback Form DE student, male.

This unease seems to have arisen from their understanding of the teacher role as a claim to expertise rather than a recognition that PAL is founded on more co-operative prinicples.

It feels strange to teach my classmates. I am speaking to people who are exceptionally bright. I worry that I am not good enough. Feedback Form DE student, male.

Going through the process itself helped to counter some negative attitudes

Once I got going I forgot about my nerves and overall it was an enjoyable and very positive experience DE student, male

### Poor engagement

Success was dependent on the engagement of most group members, without which the process was a dispiriting experience for those who were willing to undertake their assigned roles.

None of the students I talked to are very enthused about peer teaching. It’s hard to teach each other when there’s an overall feeling that people don’t want to be there and just want you to hurry up and finish so they can get on with their work. Focus Group 3 DE Student, Male.

### Desire for supervision

In focus group discussions, exploring how the process might be made more worthwhile, the commonest suggestion was the provision of faculty supervision. Students suggested that they could not be relied upon to undertake this exercise by themselves.

If there was just one presence of one person… tutor could stand in the room and maybe just monitor that it is actually going on, what they are supposed to be doing, because.. there needs to be some kind of oversight Focus Group 3, DE Student, female.

Graduate students provided an alternative perspective during focus group discussions.

I think once we introduce again the role of a tutor we are taking a huge load of the requirements off of the students…you are going to be looking to them always for guidance and approval whereas if you just do it yourself you are forced to learn a lot from each other. Focus Group 2, GE Student, female.

## Discussion

In this study we have evaluated a PAL intervention in a complex learning environment, amongst a diverse student cohort. PAL typically takes place in controlled settings, where it does not compete directly with other activities. We were interested both in how PAL worked in a new context, and how using a socio-cultural approach could add to our understanding. Our findings demonstrate that introducing PAL in the clinical environment presents challenges specific to that context. Using the lens of activity theory helped to describe student activity on clinical attachment and to highlight tensions and contradictions relating to the introduction of PAL, as well as opportunities for expansive learning.

Many evaluations of PAL programs are based on student satisfaction ratings (Bulte et al. 2007; Ten Cate 2007) and examination of assessment outcomes for evidence of learning (Burke et al. 2007; Batchelder et al. 2010). Similar approaches have been applied to the evaluation of PAL in clinical settings (Nikendei et al. 2009; Schauseil-Zipf et al. 2010). When viewed through the lens of activity theory, however,a more complex picture emerges. PAL activity in clinical environments is not undertaken in isolation. In our study the dominant student activity emerging in the clinical setting is Learning from Experts. Despite some positive comments on the teaching provided by peers, it was apparent that many students did not value the competing activity Learning with Peers in the same way. COP theory (Lave and Wenger 1991) provides an account of how students learn in the clinical environment as peripheral participants in medical practice and emphasises the importance of access to senior figures in this process. Time spent connecting with experts is about more than the learning of content; being in the company of seniors fulfils several functions, most importantly positioning learners as legitimate within the community, which is central to professional identity formation (ibid) Our students demonstrated a strong desire to learn in this way. ASA highlighted the contradiction between student “rules” or beliefs, in relation to how learning happens on clinical placements, and the nature of the PAL intervention and its desired outcome. This contradiction is specific to learning in the clinical workplace where learning is about becoming a member of a community, in contrast to learning in e.g. clinical skills labs, which is removed from the practice setting. This difference in context, made PAL less acceptable to students than has been reported in other settings (Bulte et al. 2007; Ten Cate and Durning 2007a). Students perceived that time spent on the Peer activity directly reduced time spent with Experts. Billett (2006b) has described workplace learning as being founded on the relational interdependence between the affordances, or opportunities to learn, within the workplace, and the agency of the learner, who may choose to engage with these affordances or not. In this study many students chose not to engage with an affordance, the PAL intervention, because it did not align with their personal epistemology or beliefs about learning (Billett 2009).

Our Graduate Entry students are older, and have greater experience of higher education, than the rest of the cohort. Graduates Entry students valued the possibility of Learning with Peers more than Direct Entry students. Diversity amongst medical students, in terms of age, gender and socio-economic background, is advocated for many reasons (Cohen et al. 2002) and has been proposed an educational strength (McLean et al. 2006). Many medical schools in the UK, Ireland and Australia now run parallel programmes similar to those described here, mixing older students, with prior degrees, with those entering directly from high school. Several studies have focussed on comparing the assessment outcomes (Calvert et al. 2009) and other characteristics (Hayes et al. 2004; Wilkinson et al. 2004) of these students, however, less attention has been given to the ways in which the two groups can learn from and with each other. While the difference in engagement between Graduate Entry and Direct Entry students in our study created some tension within the groups, it may also present an opportunity for expansive learning at a system level (Engestrom 2009). The graduate entry students, as peers, can provide an influential voice in promoting an activity which might otherwise be dismissed. ASA brought these underlying differences to the fore, highlighting an opportunity to capitalise on tensions and contradictions to bring about change.

The activity theory lens provided an insight into the importance of social relationships with peers amongst our students. The social discomfort associated with providing negative feedback is recognised as a barrier to effective PAL and is not specifically related to the clinical environment (Perera et al. 2010). A good network of social relationships outside the classroom has been shown to influence learning positively (Hommes et al. 2012), however, in this instance the need to “get along” successfully as a social group constrained mutual engagement and provision of feedback as a community of learners. In terms of ASA, the PAL activity was in contradiction to other activity systems operating in the lives of our students, where friendship was the dominant object.

The nature of the PAL intervention in this study might be considered a limitation. The definition of PAL emphasises the non-expert status of participants, in terms of both content and teaching skills, however, formal peer-teacher training and content control are often features of PAL in medical education. In this study there was no faculty control or vetting of the topics and content delivered in the sessions, the topics arose from cases seen on the wards and were chosen by the students as important. Apart from training in the delivery of feedback, presentation skills and in the specifics of the PAL process, our students did not have teaching skills training. This was a same level reciprocal PAL intervention with an emphasis on classmates helping each other to integrate prior learning with clinical experience. There was no evidence that any of the challenges and tensions which arose during the study were related to a lack of teaching skills amongst peer teachers, indeed many very positive comments were made in regard to the quality of presentations and material taught. Our data collection did not include any direct observations of PAL activity. We felt that the presence of a faculty member even in an observer role, would alter student behaviour. The comments made by students during focus groups in regard to supervision of PAL sessions would appear to justify our approach. Just having a faculty member in the room was perceived as being sufficiently influential as to alter student engagement.

### Implications for practice

Our findings have implications for practice. Our PAL intervention may have been too brief (seven sessions) to allow students to become accustomed to this new approach. Integration of PAL and its underpinning values into the curriculum from the outset, would allow students to develop accountability as members of a community of learners over time. This should include recognition of PAL activity in assessment. Our students’ exam focussed learning is likely to be a reflection of the nature and focus of assessments in place, which at the time this study was undertaken, did not include any evaluation of teamwork or engagement with peers. Graduate Entry students could be catalysts for expansive learning in the development such a community, challenging the views of students reluctant to take greater responsibility for their own learning. Transfer of PAL to clinical settings would be likely to be more successful if the group were already functioning as a community in that way.

Some of the social benefits of PAL, particularly relating to easing transitions, have been identified as being linked to near-peer (Lockspeiser et al. 2008), rather than same level peer, interventions such as ours. Connection with more senior learners, who can be role models for junior students, offers possibilities for professional identity formation which may not be available in same level PAL. It is possible that using students a year or two ahead of the index group would have been more successful, particularly in easing the transition to fulltime clinical placements. Other findings, such as discomfort amongst some students with the peer teacher role, are recognised features of new PAL initiatives, particularly where provision of feedback to peers is involved (Topping 1998).

Medical students will always be drawn to spending time with senior doctors and this is important for the formation of professional identity. Consideration should be given to the timing of PAL in clinical settings so that it could be more flexible and avoid taking students away from opportunities to learn from experts. Asynchronous online PAL activity might address this issue, however student engagement in these types of learning activities can also be problematic (Ellaway and Masters 2008).

## Conclusion

It is well established that qualitative research can provide richer data and deeper understanding of the learning process than other methods. Use of activity theory has been infrequent in the medical education literature (de Feijter et al. 2011; Wearn et al. 2008) and answers the call to consider what social science methodologies can offer to medical educators (Eva 2013). In this study it provided an understanding of contextual factors impacting this PAL intervention and importantly also highlighted opportunities for expansive learning that would not necessarily have been otherwise revealed. Planning learning opportunities on clinical placements, must take account of how students learn in workplaces, and the complexity of the multiple competing activity systems related to learning and social activities.
